# Localization of TRP Channels in Healthy Oral Mucosa from Human Donors

**DOI:** 10.1523/ENEURO.0328-21.2022

**Published:** 2022-12-21

**Authors:** Yalda Moayedi, Stephanie Michlig, Mark Park, Alia Koch, Ellen A. Lumpkin

**Affiliations:** 1 Department of Physiology and Cellular Biophysics, Columbia University, New York, NY 10032; 2 Department of Neurology, Columbia University, New York, NY 10032; 3 Department of Otolaryngology-Head and Neck Surgery, Columbia University, New York, NY 10032; 4 Nestlé Research, 1000, Lausanne, Switzerland; 5 Oral and Maxillofacial Surgery, New York Presbyterian, Columbia University, New York, NY 10032

**Keywords:** histochemistry, innervation, ion channels, keratinocyte, oral anatomy, TRP channels

## Abstract

The oral cavity is exposed to a remarkable range of noxious and innocuous conditions, including temperature fluctuations, mechanical forces, inflammation, and environmental and endogenous chemicals. How such changes in the oral environment are sensed is not completely understood. Transient receptor potential (TRP) ion channels are a diverse family of molecular receptors that are activated by chemicals, temperature changes, and tissue damage. In non-neuronal cells, TRP channels play roles in inflammation, tissue development, and maintenance. In somatosensory neurons, TRP channels mediate nociception, thermosensation, and chemosensation. To assess whether TRP channels might be involved in environmental sensing in the human oral cavity, we investigated their distribution in human tongue and hard palate biopsies. TRPV3 and TRPV4 were expressed in epithelial cells with inverse expression patterns where they likely contribute to epithelial development and integrity. TRPA1 immunoreactivity was present in fibroblasts, immune cells, and neuronal afferents, consistent with known roles of TRPA1 in sensory transduction and response to damage and inflammation. TRPM8 immunoreactivity was found in lamina propria and neuronal subpopulations including within the end bulbs of Krause, consistent with a role in thermal sensation. TRPV1 immunoreactivity was identified in intraepithelial nerve fibers and end bulbs of Krause, consistent with roles in nociception and thermosensation. TRPM8 and TRPV1 immunoreactivity in end bulbs of Krause suggest that these structures contain a variety of neuronal afferents, including those that mediate nociception, thermosensation, and mechanotransduction. Collectively, these studies support the role of TRP channels in oral environmental surveillance and response.

## Significance Statement

Oral mucosa experiences a myriad of environmental fluctuations during feeding, speech, and daily life which they respond to ensure normal functions. These environmental stimuli are transduced by molecular receptors, including those of the transient receptor potential (TRP) family of cation channels that reside in the membranes of mucosal cells and somatosensory neurons. The distribution of TRP channels that transduce chemical, biological, and thermal stimuli in oral mucosa is not well defined. This manuscript identifies the histologic distribution of TRP channels in healthy oral tissues and develops hypotheses about how localization lends to their essential roles in oral functions and maintenance of homeostasis.

## Introduction

Oral mucosa is poised to transduce chemosensory and somatosensory stimuli during feeding, speech, and protection against biological and chemical agents. Sensory transduction occurs through the activation of receptor molecules that detect mechanical, chemical, or thermal stimulation of tissues. The transient receptor potential (TRP) family of cation channels include molecular receptors that encode chemical, thermal, and mechanical aspects of environmental stimuli, and are important transducers of chemesthetic signals (Roper, 2014; Startek et al., 2019; Aroke et al., 2020; Luo et al., 2021; Kashio and Tominaga, 2022; Reeh and Fischer, 2022). TRPV1 is a heat-activated receptor that is also the molecular target of capsaicin, the pungent component of spicy chilies (Caterina et al., 1997; Sasase et al., 2022). TRPV3 and TRPV4 are activated by warm temperatures, chemical stimuli, and osmotic swelling (Peier et al., 2002b; Chung et al., 2004a; Vriens et al., 2004; Vogt-Eisele et al., 2007). TRPM8 is activated by cooling, menthol, and other chemicals that produce a cooling sensation (McKemy et al., 2002; Peier et al., 2002a; Yin and Lee, 2020). TRPA1, the “wasabi receptor,” is a promiscuous damage sensing receptor that is activated by noxious pungent compounds in radishes, mustard, and garlic, as well as reactive oxygen species produced during tissue stress (Bautista et al., 2005; Takahashi et al., 2008; Manolache et al., 2021; Kashio and Tominaga, 2022; Landini et al., 2022). In rodents, these TRP channels are expressed in somatosensory neurons; however, several are also reported to be expressed in epithelial cells, including the oral epithelium (Wang et al., 2011; Vandewauw et al., 2013).

With regard to oral functions, several TRP channels are important in flavor perception and pathophysiology. TRPM5 and TRPM4 are expressed in rodent and human Type II taste cells and are essential components of the signaling pathways downstream of sweet, bitter, and umami stimuli (Pérez et al., 2002; Liu and Liman, 2003; Prawitt et al., 2003; Zhang et al., 2003; Dutta Banik et al., 2018; Aroke et al., 2020). Similarly, in rodents TRP channels PKD1L3 and PKD2L1 are expressed in Type III taste cells, although their contribution to taste-cell physiology is still debated (Ishimaru et al., 2006; Horio et al., 2011; Ye et al., 2016). TRPV4 has been found to regulate Type III taste-cell differentiation in mice, loss of which results in reduced sensitivity to sour compounds (Matsumoto et al., 2019). In addition to roles in gustation, TRP channels are essential contributors to oral temperature transduction, chemesthesis, and response to injury. For example, TRPM8, TRPA1, and TRPV1 mediate sensory transduction of pungent chemicals in numerous flavor-enhancing spices, including mint, radishes, chiles, black pepper, and cinnamon (Roper, 2014). These TRP channels are also important for thermal transduction in the oral cavity (Lemon, 2021). Furthermore, TRP channel expression and activation has been linked to oral cancer cell proliferation and pain in rodents and humans (Okamoto et al., 2012; Ruparel et al., 2015; Fujii et al., 2020).

Despite the importance of TRP channels for oral functions, the localization of these channels in healthy tissues from the human oral cavity is not clear. In this study, we present an immunohistochemical analysis of TRP channels in human hard palates and tongue biopsies from healthy tissues.

## Materials and Methods

### Study enrollment criteria

Human studies were approved by the Institutional Review Board of Columbia University. Oral biopsies were collected from adult volunteers (27–45 years old, *n* = 13; Table 1). Exclusion criteria: infection, pain, oral injury, cutaneous abnormality that could interfere with safety or data interpretation, anticoagulants (e.g., aspirin, coumadin, NSAIDs), bleeding disorder, keloidal or hypertrophic scarring history, oral cancer, neurologic disease, epithelial innervation abnormality in biopsy site, a known or suspected medical or psychological condition that may affect ability to consent or to follow instructions for wound care, and an active medical condition that may affect risk of infection or healing after biopsy.

**Table 1:** Collected biopsies

Site of Biopsy	Age	Sex
Palate	27	M
Palate	28	M
Palate	30	F
Palate	35	M
Palate	40	F
Palate	43	F
Tongue	45	F
Tongue	27	F
Tongue	28	F
Tongue	28	M
Tongue	33	F
Tongue	38	F
Tongue	43	F

### Informed consent

Written informed consent was obtained by study personnel before any protocol-specific procedures. The study was conducted in accordance with the Food and Drug Administration (FDA)-approved revision of the Declaration of Helsinki, current FDA regulations, and International Conference on Harmonization guidelines.

### Tissue collection

Biopsies of either front of tongue or palate rugae were collected from each participant. Biopsy site was anesthetized (2% lidocaine with epinephrine; 1:100,000). A 4-mm punch biopsy oriented perpendicular to the specimen and punch was taken down to the submucosal layer. College pliers were used to remove the core and reveal the submucosal layer and scissors were used to free the biopsy if needed. The specimen was removed and placed in phosphate buffered saline (PBS) and pressure applied to the biopsy site. The biopsy was sutured closed if necessary and additional gauze applied. Compensation was given after biopsies were collected. Discarded human foreskin tissue was used to establish antibody concentrations and for peptide blocking experiments.

### Immunohistochemistry

**Figure 1.**
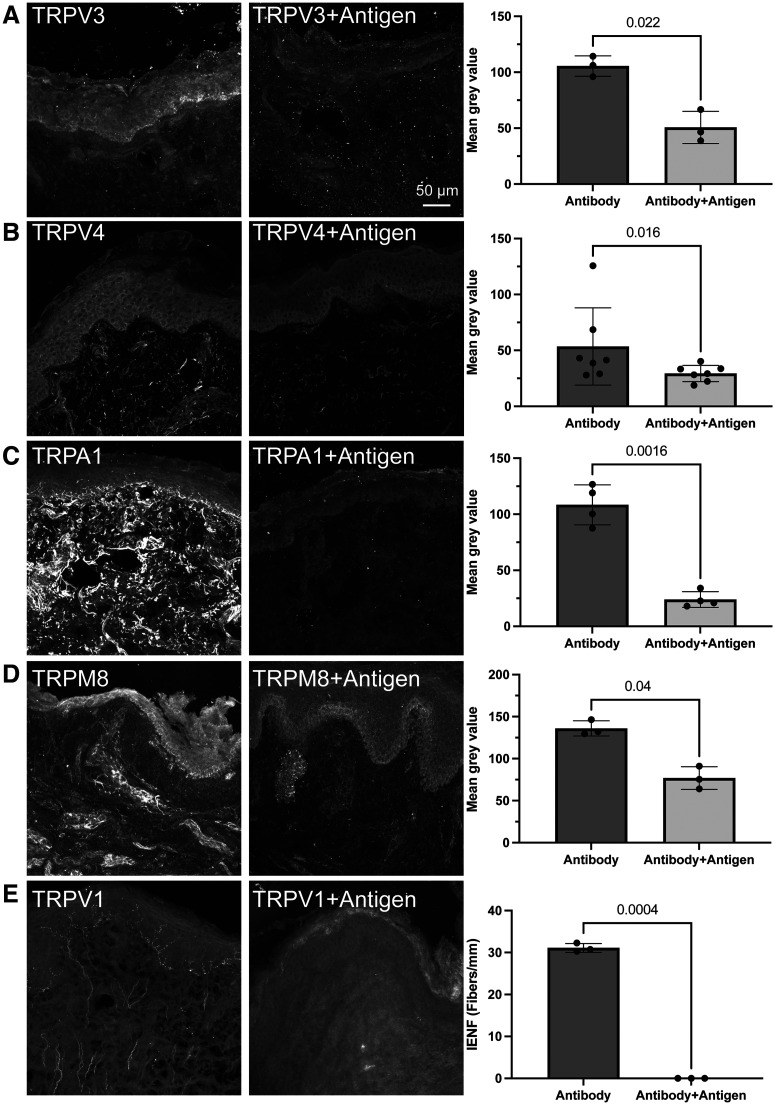
Antigen blocking shows specificity of TRP antibodies. ***A***, TRPV3 immunoreactivity was identified in the foreskin epithelium. Antigen pretreatment blocked TRPV3 immunoreactivity (*N* = 3 foreskins, paired *t* test *p* = 0.022). ***B***, TRPV4 immunoreactivity was identified in the foreskin epithelium and was diminished with antigen pretreatment (*N* = 7 foreskins, Wilcoxon matched pairs signed-rank test *p* = 0.016). Note, removal of outlier data point results in *p* = 0.02. ***C***, TRPA1 immunoreactivity was identified primarily in the dermal compartment of foreskin tissue and was completely ablated with antigen pretreatment (*N* = 4 foreskins or tongues, paired *t* test, *p* = 0.0016). ***D***, TRPM8 immunoreactivity was localized primarily to the foreskin epithelium and portions of the dermal compartment. Antigen blocking greatly diminished immunoreactivity (*N* = 3 foreskins, paired *t* test, *N* = 0.04). ***E***, TRPV1 immunoreactivity was found primarily in intraepithelial nerve fibers of the foreskin. Antigen pretreatment completely abolished TRPV1 nerve fiber immunoreactivity but not the diffuse TRPV1 immunoreactivity of epithelial cells (*N* = 3 foreskins, paired *t* test, *N* = 0.0004). All graphs show mean ± standard deviation.

Tissues were embedded (TissueTech OCT), flash frozen, and 25-μm sections were made on gelatinized slides. Slides were incubated for 30 min at 37°C, fixed with 4% paraformaldehyde (0–15 min) followed by five washes in PBS. Slides were blocked in PBS with 0.1% Triton X-100 (PBST) and 5% normal goat serum. Sections were incubated overnight with primary antibodies (Table 2) mixed in blocking buffer at 4°C. Slides were washed 3× in PBST and incubated with secondary antibody in blocking solution for 1–2 h, then washed 5× in PBS and mounted in Fluoromount-G with DAPI. Specimens were imaged with a laser scanning confocal microscope equipped with 40× (NA 1.3) and 20× (NA 0.8) lenses with a Z-step size of 1 μm through the entire depth of each section (25 μm). Antibody concentrations and staining parameters were optimized on foreskin tissue. In antigen blocking experiments, primary antibody cocktail was preincubated with 5× protein antigen concentration relative to antibody concentration 30 min at room temperature before incubation with sections. Results of antigen retrieval experiments performed on discarded foreskin tissue are shown in Figure 1. For each biomarker, 2–4 independent samples from front of tongue and hard palate rugae were tested.

**Table 2:** Antibodies used in this study

Antibody	Supplier	Catalog #	Lot	Dilution	RRID
Mouse anti-Keratin 20	Abcam	Ab854	GR157163-5	1:100	AB_2133708
Chicken anti-Neurofilament-Heavy	Abcam	Ab4680	GR310109-11	1:5000	AB_304560
Mouse anti-βIII tubulin (Tuj1)	Neuromics	MO15013	402154 and 402360	1:100	AB_2737114
Moue anti-CD45	Abcam	Ab781	GR3233952-8	1:100	AB_306098
Rabbit anti-TRPA1	Alomone Labs	ACC-037	ACC037AN17	1:500	AB_2040232
Rabbit anti-TRPV1	Abcam	Ab3487	GR3219961-4	1:500	AB_2209009
Rabbit anti-TRPM8	Alomone Labs	ACC-049	ACC049AN15	1:100	AB_2040254
Rabbit anti-TRPV4	Lifespan Bio	LS-A8583	61861	1:100	AB_592927
Rabbit anti-TRPV3	Alomone Labs	ACC-033	ACC033AN02	1:100	AB_2040261

### Quantification of immunohistochemistry

All quantification was performed in ImageJ (version 2.3.0/1.53f). Efficiency of TRPV1 antigen blocking was quantified by calculating the number of TRPV1+ intraepithelial nerve fibers (IENF; defined as TRPV1+ nerve endings crossing the basement membrane of the epithelium) divided by the length of basement membrane in each image. Three images were taken from each of three samples of foreskin and averaged per sample. Efficiency of antigen blocking for TRPV3, TRPV4, TRPM8, and TRPA1 was analyzed by calculating the average mean gray value of three regions of epithelium (TRPV3, TRPV4, TRPM8) or lamina propria (TRPA1) from each of three images per foreskin sample.

To calculate percent of epithelial depth occupied by TRPV3 and TRPV4, thick lines (50 μm) were applied to each image from the outside of the epithelium through the epithelium and past the basement membrane. Profiles were plotted to extract the mean gray value for each point along the line and used to determine the start and ends of DAPI staining or immunoreactivity. Start of staining or DAPI was defined as 50% of the first peak and end of staining or DAPI was defined as 50% drop from the last peak. Epithelial depth was calculated from the first incidence of DAPI staining on the outer edge of the cornified layer of epithelium until the drop-off in DAPI staining at the basement membrane border. Percent of epithelium with TRPV3 and TRPV4 immunoreactivity was calculated as the percent of epithelial depth occupied with 0 being the edge of the cornified layer of epithelium and 100 being the drop off point at the basement membrane border.

To calculate the density of TRPA1 immunoreactivity, six to seven images of palate or tongue were thresholded using default settings in ImageJ. The epithelial and lamina propria compartments were traced and the area fraction of TRPA1 immunoreactivity extracted. The proportion of CD45+ cells with TRPA1 immunoreactivity were manually counted in ImageJ.

### Statistical analysis

Statistics were performed using Prism 9 (GraphPad version 9.4.1). All data were tested for normality and statistical tests chosen accordingly. Data were tested for normality and either paired t test were Wilcoxon test applied.

## Results

To directly compare immunoreactivity of a panel of TRP channels in human oral cavity, biopsies of hard palate rugae or tongue papillae were collected from healthy adult volunteers. Antibodies against TRP channel targets were first optimized on human foreskin tissue, and then tested on at least two independent samples of both tongue and hard palate rugae for each probe. Antigen blocking experiments were performed to test the specificity of each antibody for its antigen (Fig. 1). Antigen specificity was tested for each antibody in at least three independent replicates.

**Figure 2.**
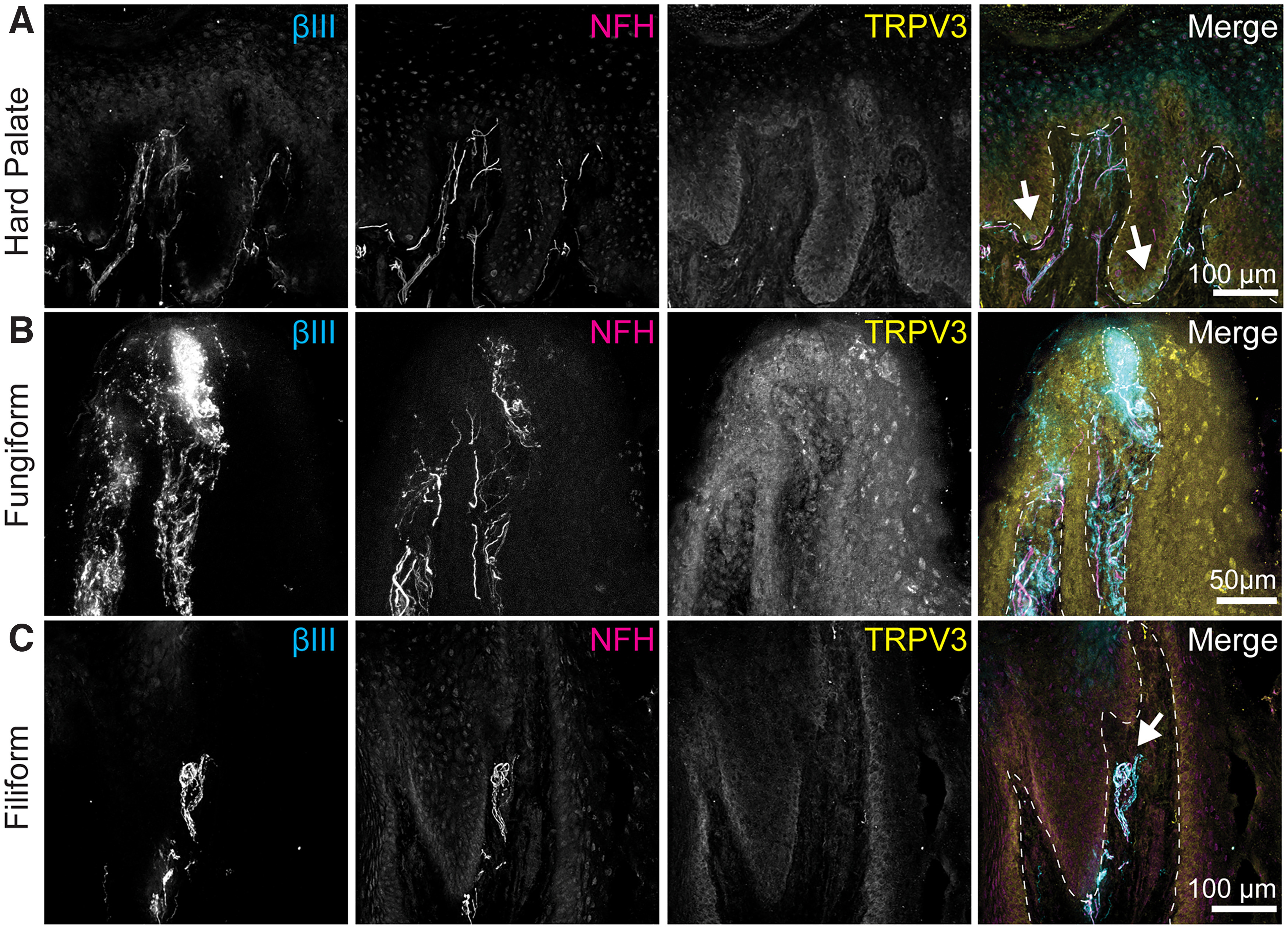
TRPV3 is expressed in basal layers of oral epithelium. Left column, Tuj1 anti-βIII tubulin (all afferent neurons). Second column, Anti-NFH antibody (myelinated neurons). Third column, Anti-TRPV3 antibody. Right column, Merge with TRP immunoreactivity in yellow, βIII immunoreactivity in cyan, NFH immunoreactivity in magenta. Dashed line indicates epithelia-lamina propria border. ***A***, TRPV3 expression was found in basal epithelial layers of the hard palate as well as in the lamina propria. White arrows denote areas where Merkel cells typically concentrate (Moayedi et al., 2021). Note that Merkel cell afferents are visible with βIII tubulin staining in cyan. ***B***, TRPV3 immunoreactivity was dispersed throughout fungiform epithelium. ***C***, TRPV3 expression was found in basal epithelium of filiform papilla. A TRPV3 negative end bulb of Krause (arrow) is found within the lamina propria of the filiform papilla visualized with expression of βIII tubulin and NFH.

Expression of TRPV3 and TRPV4, two channels that show high expression in epithelial cells, was examined. In oral tissue, TRPV3 immunoreactivity was found primarily in the basal epithelial layers of both hard palate and tongue mucosa (Fig. 2). In the hard palate, TRPV3 localization in the basal epithelium overlapped with regions where Merkel cells are typically found (Fig. 2*A*; Moayedi et al., 2021). In the hard palate, TRPV3 immunoreactivity extended from 70.87 ± 11.32% to 99.74 ± 3.38% of epithelial thickness (mean ± SD, *N* = 3 images). In the tongue, TRPV3 immunoreactivity extended from 67.72 ± 23.98% to 101.92 ± 6.76% of epithelial thickness (mean ± SD, *N* = 4 images; Fig. 2*B*,*C*). TRPV3 immunoreactivity was undetectable in oral neurons in this study.

**Figure 3.**
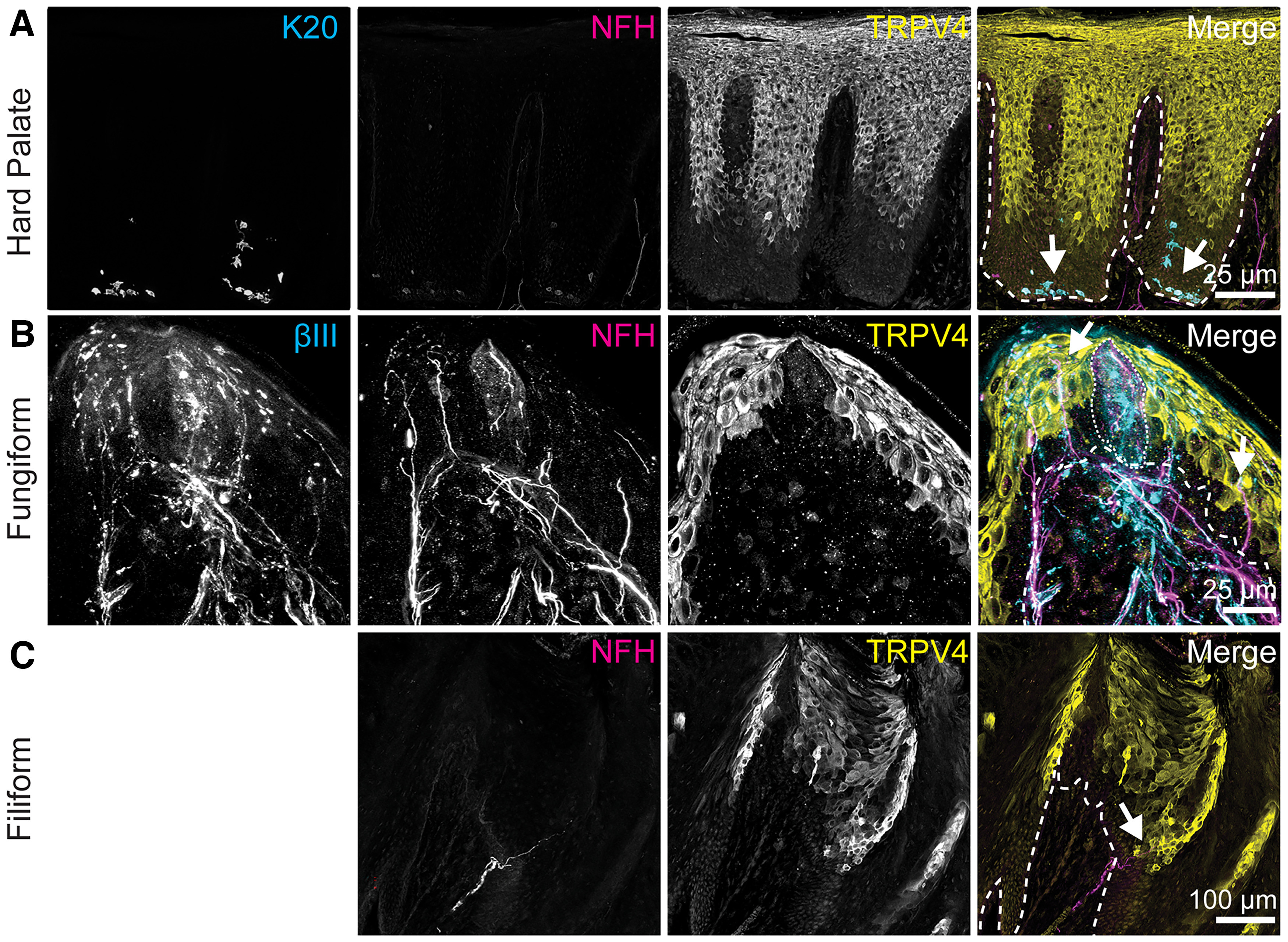
TRPV4 is expressed in apical keratinocytes. Left column, Anti-K20 (Merkel cells) or Tuj1 anti-βIII tubulin (all afferent neurons). Second column, Anti-NFH antibody (myelinated neurons). Third column, Anti-TRPV4 antibody. Right column, Merge with TRP immunoreactivity in yellow, βIII or K20 immunoreactivity in cyan, NFH immunoreactivity in magenta. Dashed line indicates epithelia-lamina propria border. Dotted line indicates location of taste bud. ***A***, Expression of TRPV4 was found in the upper layers of the hard palate epithelium. TRPV4 does not appear to be expressed in Merkel cells (white arrows). ***B***, TRPV4 was expressed in apical keratinocytes in fungiform papillae epithelium, but excluded from taste bud (white dotted line). Several nearby NFH+ and βIII+ neuronal afferents extended into TRPV4-expressing epithelial layers (white arrows). ***C***, TRPV4 was expressed in apical epithelial cells of filiform papillae. An intraepithelial NFH+ fiber (white arrow) in close association with TRPV4+ epithelial cells is shown.

TRPV4 antibodies showed strong immunoreactivity in both tongue and hard palate (Fig. 3). In hard palate, immunoreactivity was localized in outer epithelium of hard palate mucosa and did not overlap with Merkel cells (Fig. 3*A*). TRPV4 immunoreactivity in the hard palate extended from 4.65 ± 2.86% to 86.11 ± 5.07% of epithelial thickness (mean ± SD, *N* = 5 images). In the tongue, TRPV4 immunoreactivity was also identified in outer epithelial layers (Fig. 3*B*,*C*); however, it was specifically excluded from taste buds (Fig. 3*B*). In lingual epithelium, TRPV4 immunoreactivity was present between 7.84 ± 1.21% to 78.57 ± 7.62% of epithelial thickness (mean ± SD, *N* = 4 images). Neuronal endings innervating epithelial mucosa surrounding the taste buds, including NFH+ and NFH– afferents, were frequently observed extending into TRPV4+ epithelial layers (4/4 taste buds from two biopsies). In filiform papilla, intraepithelial nerve fibers were identified extending into TRPV4+ lamina (2/2 filiform papillae from two biopsies; Fig. 3*C*).

**Figure 4.**
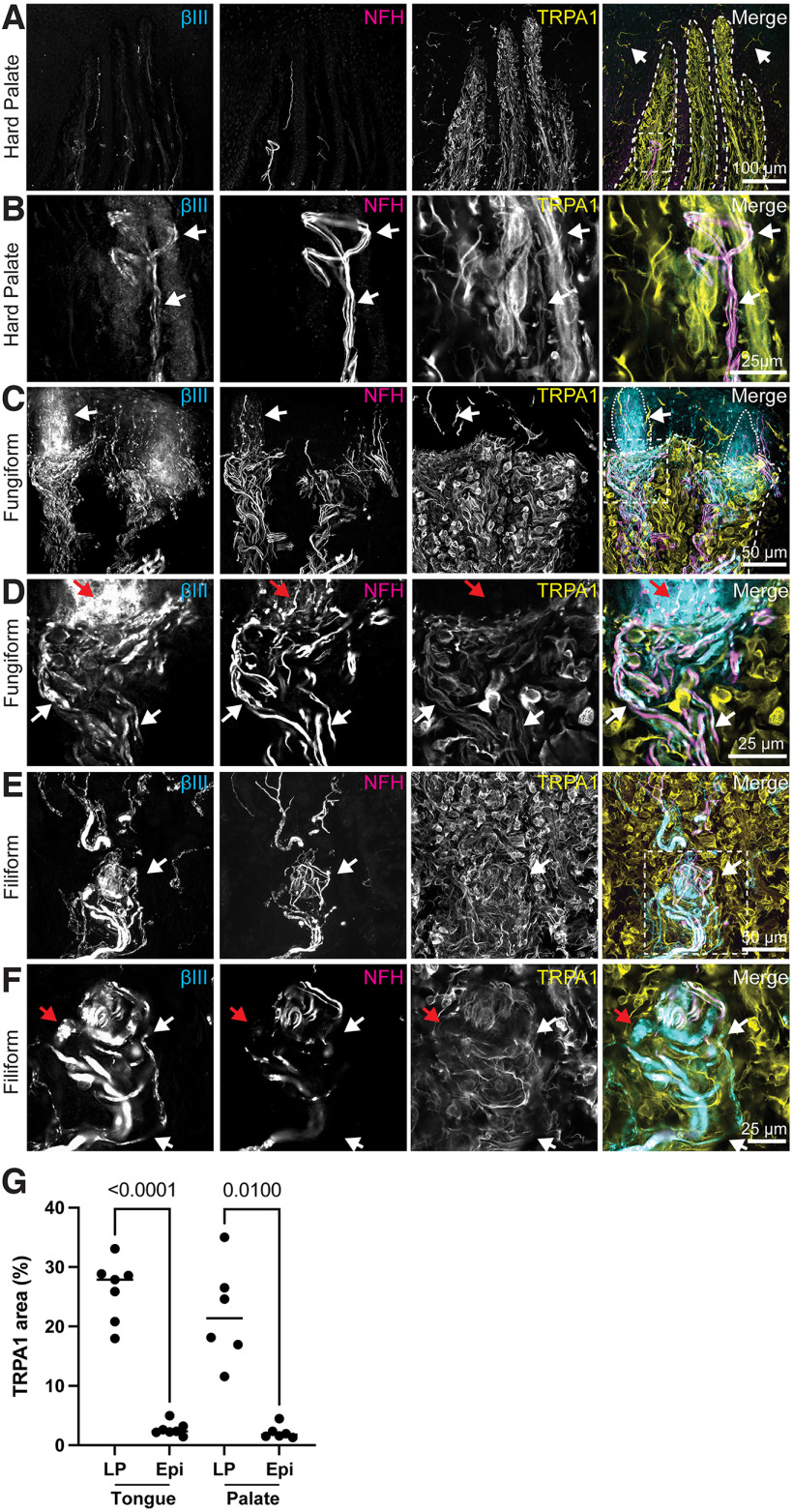
TRPA1 immunoreactivity is found in lamina propria, epithelium, and neuronal afferents. Left column, Tuj1 anti-βIII tubulin (all afferent neurons). Second column, Anti-NFH antibody (myelinated neurons). Third column, Anti-TRPA1 antibody. Right column, Merge with TRP immunoreactivity in yellow, βIII immunoreactivity in cyan, NFH immunoreactivity in magenta. Dashed line indicates epithelia-lamina propria border. Dotted line indicates location of taste buds. Dashed box indicates area of detailed, single focal plane images (***B***, ***D***, ***F***). ***A***, TRPA1 was broadly expressed throughout lamina propria cells of the hard palate as well as sparsely in the epithelium (white arrows). Co-expression of TRPA1 and the immune cell marker CD45 is shown in Extended Data Figure 4-1. A bundle of NFH+ and NFH– neuronal fibers was identified in this peg (white dashed box). White dashed box shows region in ***B***. ***B***, A higher magnification, single optical plane (1 μm) of the neuronal fibers in ***A*** showed TRPA1 expression overlapping with neuronal fibers (white arrows). ***C***, TRPA1 was broadly expressed throughout lamina propria cells in fungiform papillae as well as in some epithelial cells near taste buds (white arrow). White dashed box shows region in ***D***. Expanded views of taste buds are shown in Extended Data Figure 4-2. ***D***, A single optical plane (1 μm) from ***C*** is shown. TRPA1 expressing fibers overlapped with βIII + neuronal fibers (white arrows). βIII + neuronal endings that extended into epithelium did not express TRPA1 (red arrow). ***E***, TRPA1 was broadly expressed throughout lamina propria cells in filiform papillae and in an end bulb of Krause (white arrow). White dashed box shows region in ***F***. ***F***, Single optical plane (1 μm) from ***E*** shows neuronal afferents expressing TRPA1 (white arrows). Larger afferents appeared to be negative for TRPA1 (red arrow). ***G***, TRPA1 immunoreactivity in the lamina propria (LP) and epithelium (Epi) of tongue (*N* = 7 images from 3 biopsies) and hard palate (*N* = 6 images from 2 biopsies) was quantified. Lamina propria has significantly higher TRPA1 immunoreactivity compared with epithelium (Brown–Forsythe ANOVA *p* < 0.0001, Dunnett’s T3 multiple comparisons test). Line indicates median.

TRPA1 antibodies showed robust immunolocalization in the hard palate and tongue, with the highest density in lamina propria cells (Fig. 4*A–F*). Densities of TRPA1 immunoreactivity was compared between epithelial and lamina propria compartments, and found to be significantly lower in the epithelium in both tongue and hard palate samples (Fig. 4*G*). Sparse TRPA1+ epithelial cells were also found with dendritic morphologies consistent with immune cells (Fig. 4*A*,*C*, white arrows). Co-staining with an antibody against CD45, a marker for immune cells, revealed that many TRPA1+ lamina propria and epithelial cells were CD45+ (Extended Data Fig. 4-1). TRPA1 immunoreactivity overlapped with 54 ± 9.1% of CD45+ cells (*N* = 5 images from two biopsies). To test whether neuronal processes were also TRPA1+, we analyzed single optical planes for co-labeling of TRPA1 and βIII-tubulin, a cytoskeletal marker labeling all peripheral somatosensory neurons. This method identified neuronal afferents that were TRPA1+ in the lamina propria of hard palate (Fig. 4*B*, white arrows). In the fungiform papillae, we found TRPA1+ nerve fibers in the plexus below the taste bud (Fig. 4*D*, white arrows). Interestingly, neurons innervating the taste bud were not clearly labeled with TRPA1 antibody (Fig. 4*D*, red arrows; Extended Data Fig. 4-2). TRPA1 immunoreactive fibrous structures were identified in the taste buds, but these did not clearly co-localize with neuronal markers, indicating that they are non-neuronal and potentially processes of immune cells or other resident-epithelial cells (Extended Data Fig. 4-2). We next analyzed end bulbs of Krause in the lamina propria of filiform papillae (Fig. 4*E*,*F*, white arrow). TRPA1 immunoreactivity was present in some NFH– fibers of the end bulb of Krause (Fig. 4*F*, white arrow). Large neuronal fibers that were not immunoreactive to TRPA1 antibodies also contributed to the end bulb of Krause (Fig. 4*F*, red arrow). Collectively, these data show that TRPA1 immunoreactivity is present in oral lamina propria cells, immune cells, and subsets of neurons.

10.1523/ENEURO.0328-21.2022.f4-1 Extended Data Figure 4-1 TRPA1 immunoreactivity co-expresses with CD45 in tongue and hard palate rugae. Left column, NFH antibody (myelinated neurons). Second column, Anti-TRPA1 antibody. Third column, Anti-CD45 antibody (immune cells). Right column, Merge with TRP immunoreactivity in yellow, CD45 immunoreactivity in cyan, NFH immunoreactivity in magenta. Dashed line indicates epithelial-lamina propria border. ***A***, TRPA1 was broadly expressed throughout lamina propria cells and some cells in epithelial layer of tongue. Co-expression of TRPA1 and CD45 was identified in some epithelial cells (yellow arrows). TRPA1+ cells that did not co-express CD45 (white arrows) were also found. Red box shows region in ***B***. ***B***, A higher magnification view of ***A***. ***C***, An expanded view of ***B***. Magenta arrow denotes CD45+ cell that does not overlap with TRPA1 immunoreactivity. ***D***, TRPA1 shows a similar pattern of expression in the hard palate as in tongue. Both cells that co-expressed CD45 (yellow arrows) and those that did not (white arrows) were identified. ***E***, Higher magnification view of ***D***. ***F***, Expanded view of ***E***. Magenta arrow denotes CD45+ cell that does not overlap with TRPA1 immunoreactivity. Download Figure 4-1, TIF file.

10.1523/ENEURO.0328-21.2022.f4-2 Extended Data Figure 4-2 TRPA1 immunoreactivity does not colocalize with neuronal fibers in the taste bud. Expanded views of taste buds in Figure 4 are shown. Left column, Anti-TRPA1 antibody. Second column, βIII tubulin antibody (all neurons). Third column, Neurofilament Heavy antibody (myelinated neurons). Right column, Merge with TRP immunoreactivity in yellow, βIII immunoreactivity in cyan, NFH immunoreactivity in magenta. ***A***, TRPA1 immunoreactivity was identified in large-diameter fibrous processes in the taste bud (purple arrows). This expression did not colocalize with βIII-tubulin or NFH, suggesting that it is non-neuronal. Adjacent to the taste bud, a small fiber was identified with co-expression of βIII-tubulin (white arrow), suggesting it is neuronal. ***B***. A second taste bud was assessed. Similarly, large processes that were TRPA1 immunoreactive did not co-express βIII-tubulin or NFH (purple arrows); however, small processes were identified near the basement membrane with colocalization of βIII-tubulin and TRPA1 immunoreactivity (white arrow). Download Figure 4-2, TIF file.

**Figure 5.**
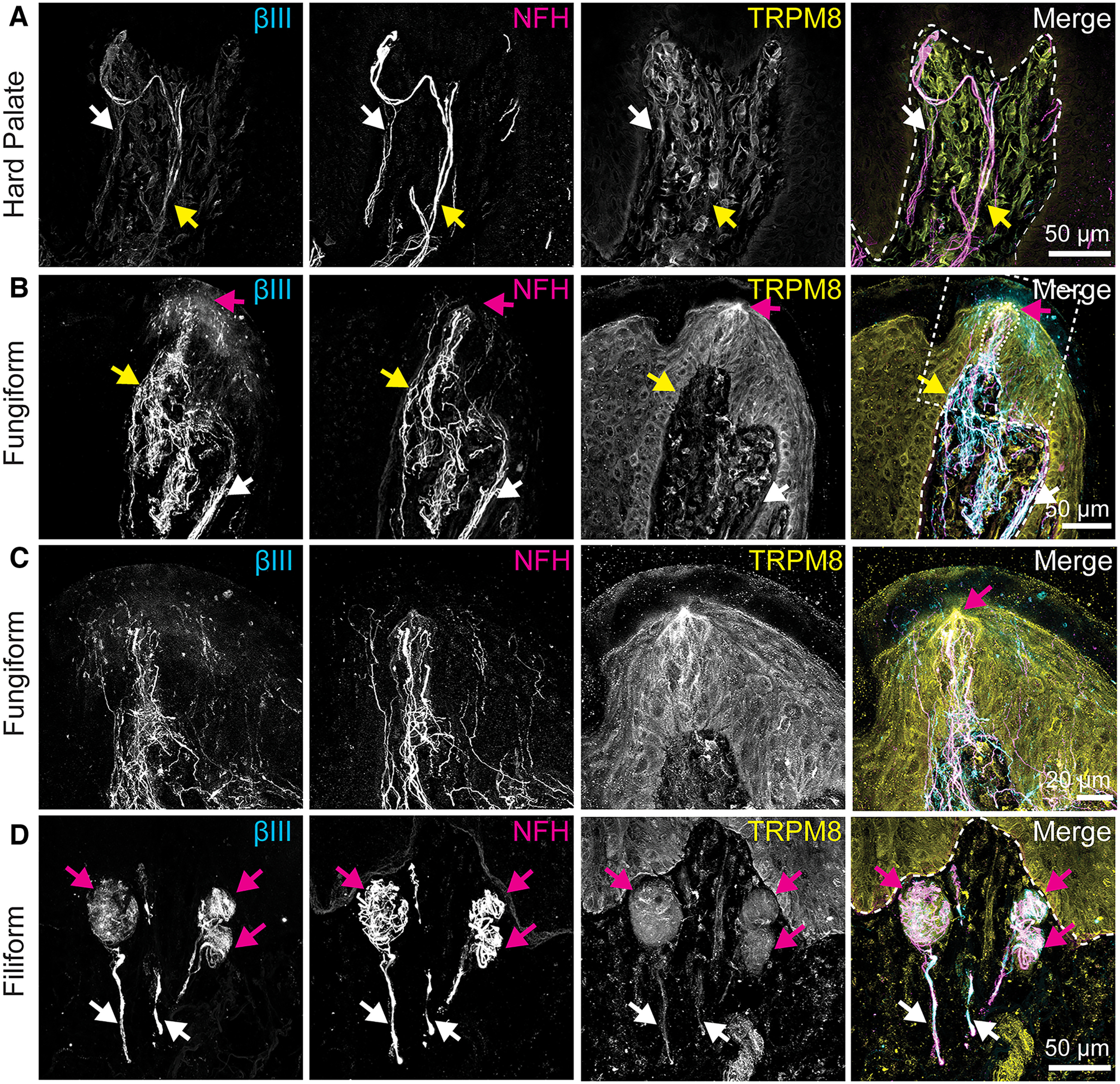
TRPM8 immunoreactivity is found throughout lamina propria, epithelium, and in neurons in oral epithelia. Left column, Tuj1 anti-βIII tubulin (all afferent neurons). Second column, Anti-NFH antibody (myelinated neurons). Third column, Anti-TRPM8 antibody. Right column, Merge with TRP immunoreactivity in yellow, βIII immunoreactivity in cyan, NFH immunoreactivity in magenta. Dashed line indicates epithelia-lamina propria border. Dotted line indicates location of taste bud. ***A***, In hard palate epithelium, TRPM8 immunoreactivity was found in lamina propria cells and some neuronal afferents. White arrow denotes TRPM8 expression in neuronal fibers. Yellow arrow denotes TRPM8 negative fibers. ***B***, In tongue mucosa, TRPM8 immunoreactivity was found in epithelium and lamina propria cells. Within taste bud, a higher concentration of TRPM8 was found near the taste pore (magenta arrow). Some neuronal afferents expressed TRPM8 (white arrow), while others were TRPM8 negative (yellow arrow). Dashed box indicates region in ***C***. ***C***, Expanded view of ***B***. Magenta arrow indicates TRPM8 immunoreactivity near the taste pore. ***D***, In filiform papillae, TRPM8 was expressed in epithelium and lamina propria cells. Magenta arrows indicate end bulbs of Krause with expression of TRPM8 in some neuronal fibers within bulbs. White arrows denote TRPM8+ neuronal afferent leading into end bulb of Krause.

TRPM8 immunoreactivity was next analyzed (Fig. 5). This antibody showed broad, low-level immunoreactivity throughout epithelial and lamina propria cells with higher signal in some neuronal afferents and lamina propria cells. In the hard palate, TRPM8 immunoreactivity was widespread in lamina propria cells (Fig. 5*A*). Within palate rugae, we identified TRPM8+ (Fig. 5*A*, white arrows) as well as TRPM8– neuronal fibers (Fig. 5*A*, yellow arrows). In fungiform papillae (Fig. 5*B*), diffuse TRPM8 immunoreactivity in the mucosa and cells of the lamina propria was found. TRPM8 immunoreactivity concentrated in the taste bud region, near the taste pore (Fig. 5*B*, magenta arrow, 2/2 total taste buds observed). An expanded view of the taste bud (Fig. 5*C*) shows immunoreactivity near the taste pore. Note that there is not complete overlap between βIII-tubulin and TRPM8 immunoreactivity. This could indicate that TRPM8 is not expressed in neuronal fibers in this region, or it could be because of membranous TRPM8 localization that does not overlap with cytoskeletal proteins in neuronal endings. Within the lamina propria of fungiform papillae, neuronal bundles were identified with TRPM8 immunoreactivity (Fig. 5*B*, white arrow), as well as TRPM8– neuronal fibers (Fig. 5*B*, yellow arrow). TRPM8 antibody showed similar localization in tongue filiform papillae, with broad immunoreactivity in the epithelium and lamina propria, as well as in some neuronal bundles (Fig. 5*D*). Within all end bulbs of Krause observed in this study (Fig. 5*D*, magenta arrows, five total), TRPM8 immunoreactivity overlapped with neuronal markers, consistent with speculation that end bulbs might be cold receptors (Ham, 1950). We also identified TRPM8-immunoreactive neuronal fibers within the lamina propria (Fig. 5*D*, white arrows).

**Figure 6.**
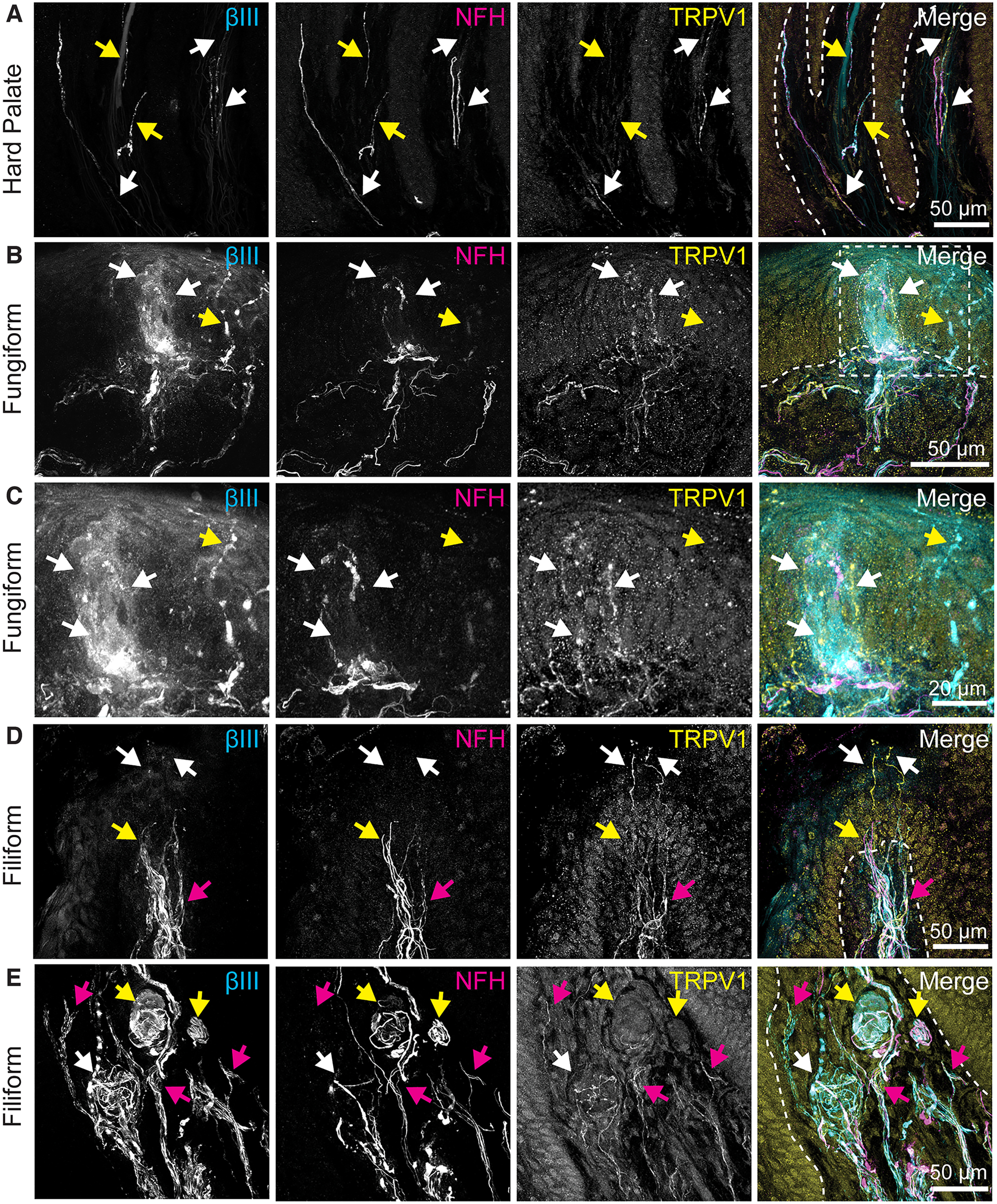
TRPV1 immunoreactivity is present in neuronal subsets of oral mucosa. Left column, Tuj1 anti-βIII tubulin (all afferent neurons). Second column, Anti-NFH antibody (myelinated neurons). Third column, Anti-TRPV1 antibody. Right column, Merge with TRP immunoreactivity in yellow, βIII immunoreactivity in cyan, NFH immunoreactivity in magenta. Dashed line indicates epithelia-lamina propria border. Dotted line indicates location of taste bud. ***A***, TRPV1 immunoreactivity was found in some neuronal afferents in the hard palate (white arrows). Nearby NFH+ TRPV1– neuronal fibers (yellow arrows) were also found. ***B***, TRPV1 was identified in intragemmal fibers in fungiform papillae (white arrows). Nearby extragemmal fibers were negative for TRPV1 immunoreactivity (yellow arrow). ***C***, Expanded view of ***B***. ***D***, TRPV1 was found in intraepidermal nerve fibers in apical tips of filiform papillae (white arrows), as well as a bundle of TRPV1 fibers in the lamina propria (magenta arrow). Nearby NFH+ TRPV1– fibers were also found (yellow arrow). ***E***, End bulbs of Krause were found in lamina propria of filiform papilla. TRPV1+ fibers were found throughout lamina propria (magenta arrows). One end bulb of Krause in this bundle had a low density of NFH+ fibers and a high density of TRPV1+ fibers (white arrow). Two additional end bulbs of Krause had a high density of NFH+ fibers and a low density of TRPV1+ fibers (yellow arrows).

Lastly, we analyzed immunoreactivity of TRPV1 (Fig. 6). In the hard palate, TRPV1+ neuronal fibers extended into the lamina propria pits of epithelial pegs (Fig. 6*A*, white arrows). NFH+, TRPV1– afferents were found nearby (Fig. 6*A*, yellow arrows). TRPV1 immunoreactivity was often observed in epithelial cells (Fig. 6*B–E*); however, this fluorescence reflects nonspecific staining, as it remained in antigen blocking controls (Fig. 1*E*). In fungiform papillae, TRPV1 expression was identified in intragemmal fibers of the taste bud, but not in nearby extragemmal fibers (Fig. 6*B,C*, white and yellow arrows, respectively, 2/2 taste buds observed). In filiform papillae, TRPV1+ intraepidermal nerve fibers were found (Fig. 6*D*, white arrows). Nearby, NFH+, TRPV1– neurons were also present (Fig. 6*D*, yellow arrow). Within the lamina propria of filiform papillae TRPV1+ fibers were frequently present (Fig. 6*E*, magenta arrows). Most end bulbs of Krause in filiform papilla had high densities of NFH+ neuronal endings; these were largely absent of TRPV1+ labeling (Fig. 6*E*, yellow arrows). Interestingly, a subset of end bulbs had TRPV1+ neuronal immunoreactivity but with comparatively few NFH+ fibers (Fig. 6*E*, white arrows, 3/11 total end bulbs of Krause). These data demonstrate that there is heterogeneity in neuronal composition of end bulbs of Krause.

## Discussion

**Figure 7.**
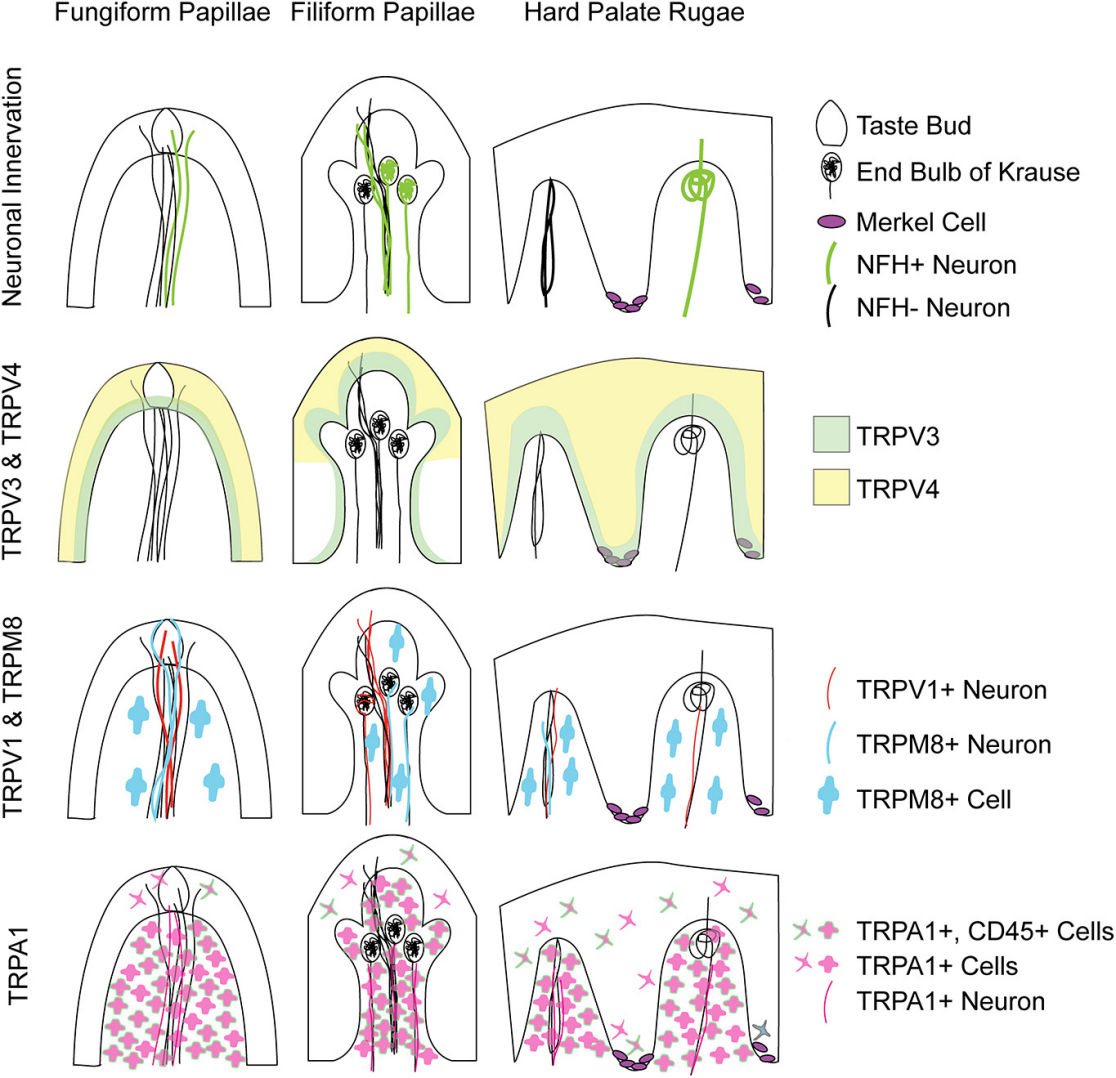
Summary of TRP channel expression in oral tissues. Distribution of TRP immunoreactivity in human tongue and hard palate is shown.

TRP channels are widely expressed in the oral cavity and subserve a variety of functions including flavor transduction, somatosensation, and stress responses. Despite this, expression of TRP channels in human oral mucosa is not well defined. In this work, we describe expression of somatosensory TRP channels in tongue and hard palate of healthy human donors (Fig. 7). We identified varied patterns of expression in epithelium, lamina propria, and neuronal afferents.

Oral epithelial cells display rapid turnover and fast wound healing rates because of intrinsic differences in oral stem cells and keratinocytes (Andl et al., 2016; Iglesias-Bartolome et al., 2018). During homeostatic turnover and wound healing, epithelial barrier integrity must be maintained to prevent infection from oral bacterium; thus, molecules involved in barrier integrity and epithelial maintenance are particularly important in oral epithelium. TRPV3 and TRPV4 are warm activated channels expressed in keratinocytes and have essential roles in epidermal development and homeostasis (Chung et al., 2004b). TRPV3 has been shown to be important for keratinocyte development and hair morphogenesis while TRPV4 is essential for skin barrier formation in mice and humans (Denda et al., 2007; Cheng et al., 2010; Sokabe et al., 2010; Kida et al., 2012; Lin et al., 2012; Akazawa et al., 2013; Blaydon and Kelsell, 2014). In the oral cavity, TRPV3 and TRPV4 were expressed primarily in epithelium with inverse distributions. TRPV3 immunoreactivity was observed throughout basal layers of epithelium in both the tongue and hard palate, similar to previous findings in mice and humans (Xu et al., 2006). In contrast, TRPV4 immunoreactivity was found in apical layers of oral cavity epithelium, and was excluded from taste cells and Merkel cells. This expression pattern in outer keratinocyte layers in oral tissues is consistent with expression in palmar keratinocytes (Blaydon and Kelsell, 2014). The expression of TRPV3 and TRPV4 was consistent with previous studies in rats showing that oral epithelia respond to TRPV3 and TRPV4 agonists including camphor, 4α-phorbol-12,13 didecanoate (4α-PDD), and 2-aminoethoxydiphenyl borate (2-APB; Wang et al., 2011). Based on expression patterns and known functions of TRP channels, we can build hypotheses on the roles in oral tissues. TRPV3 likely plays an important role in oral epithelial growth and renewal, particularly after damage (Aijima et al., 2015). TRPV4 is likely playing a role in oral barrier formation and may mediate inflammatory signaling and pain after tissue damage (Sokabe et al., 2010; Moore et al., 2013; Rajasekhar et al., 2017). Furthermore, as TRPV4 responds to shear stress, it may also function in cell signaling in response to epithelial stretch (Rajasekhar et al., 2017).

TRPA1 is a key damage sensor in many organs and tissues (Talavera et al., 2020; Naert et al., 2021). Consistent with this, TRPA1 immunoreactivity was identified predominantly in cell types that are poised to report tissue injury including cells in the lamina propria and immune cells. The widespread expression of TRPA1 in the lamina propria of hard palate and tongue suggests that it is expressed broadly in oral fibroblasts. TRPA1 is functionally expressed in human dental fibroblasts, suggesting that this is a conserved TRPA1 pattern of expression in the oral cavity (El Karim et al., 2011). TRPA1 expression within oral lamina propria provides an optimal localization to play a role in remodeling because of tissue damage. Subpopulations of TRPA1+ cells in lamina propria and epithelium colocalized with CD45, a well-established marker of immune cells, indicating that TRPA1+ is expressed in subsets of immune cells of oral mucosa. The widespread expression of TRPA1 is consistent with a role of TRPA1 in inflammation in oral cavity. Expression in immune cells and fibroblast positions this channel to signal the presence of noxious compounds or tissue damage.

In addition to roles in responding to cellular damage, TRPA1 is activated by pungent compounds, like wasabi and allicin, and plays a role in chemesthesis during flavor construction (Talavera et al., 2020). TRPA1 immunoreactivity was observed in afferents innervating both the tongue and hard palate, largely excluding large diameter neuronal endings. Expression in neuronal afferents confers these neuronal endings with the ability to detect pungent compounds in the mouth, or to take part in pain, itch, and thermal signal transduction.

Temperature sensation in oral tissues is an important aspect of flavor construction. TRPM8 is a cold and menthol-activated receptor that is essential for cold and warm sensations (Moore et al., 2018; Paricio-Montesinos et al., 2020). Neuronal immunoreactivity to TRPM8 was identified in hard palate and tongue mucosa. In the filiform papillae of the tongue, TRPM8+ neuronal fibers were also found within end bulbs of Krause, indicating that these structures may include cold sensitive afferents as initially theorized (Ham, 1950). TRPM8 immunoreactivity was also found to concentrate around the taste bud in fungiform papillae of the tongue, similar to previous findings in rodents (Abe et al., 2005; Dhaka et al., 2008). Expression around fungiform papilla taste buds is likely to contribute to chemesthesis of flavors of compounds like menthol and eucalyptol as well as transduction of cold sensations. In addition to neuronal localization, we identified TRPM8 immunoreactivity in oral fibroblasts, consistent with previous findings in human oral tissues (El Karim et al., 2011). Here, TRPM8 may take part in remodeling in response to chemical or thermal activation.

TRPV1 is expressed in nociceptive afferents and epithelial cells and is responsive to capsaicin, heat, pH, and histamine, among other compounds (Moore et al., 2018). TRPV1 activating compounds are particularly important in regards to oral function as they mediate flavor construction, oral homeostasis, and response to pathogens and injury. Epithelial expression of TRPV1 has been shown previously in skin, where it plays a role in keratinocyte migration, and epidermal barrier integrity (Denda et al., 2001; Blaydon and Kelsell, 2014). Oral epithelia respond to capsaicin, suggesting that TRPV1 expression is functional in oral keratinocytes (Wang et al., 2011). We found light immunoreactivity for the TRPV1 antibody in the epithelium of tongue and hard palate; however, this labeling persisted despite antigen blocking and thus we could not confirm these findings. Neuronal TRPV1 immunoreactivity was widespread in the oral cavity and specific to the antigen. We identified TRPV1+ free nerve endings in the tongue and hard palate, including intraepithelial nerve fibers. These neurons would be suspected to take part in nociception, temperature sensation, and chemesthesis. In fungiform taste buds, we identified TRPV1 immunoreactivity in intragemmal fibers of the taste bud, but not in extragemmal fibers, consistent with findings in rats (Ishida et al., 2002). These intragemmal fibers are ideally positioned to take part in flavor construction by transducing temperature and spiciness of foods. Within filiform papillae of the tongue, TRPV1 immunoreactivity was surprisingly identified in neuronal fibers associated with end bulbs of Krause. These findings suggest a previously unappreciated diversity in the population of neurons within end bulbs of Krause, having both myelinated, likely mechanosensory neurons intermingled with unmyelinated TRPV1+ and TRPM8+ populations that may take part in thermosensation. We also noted diversity in end bulbs of Krause compositions within a single filiform papilla, with some having dense NFH+ myelinated afferents while others having fewer myelinated afferents and higher unmyelinated, TRPV1+ afferents. The finding of independent neuronal afferent subtypes within a single corpuscle are reminiscent of findings in Meissner’s corpuscles, where two distinct populations of mechanoreceptors have been found in mice, and where both myelinated and unmyelinated neurons have been identified in humans (Cauna, 1956; Neubarth et al., 2020). Future studies are required to parse out differences in end bulbs of Krause populations and to better understand the functions of these structures in somatosensation.

TRPV1 expressing neurons play important roles in nociception, inflammatory pain, and neuropathic pain (Moore et al., 2018). Patients with burning mouth syndrome (BMS) have an increase in both the presence of TRPV1+ nerve fibers and in epithelial TRPV1 expression (Yilmaz et al., 2007; Borsani et al., 2014). This suggests that expression of TRPV1 could be linked to the pathogenesis of BMS. Future studies should be performed investigating whether TRPV1 expression is upregulated in particular ending types, such as intraepidermal nerve fibers or end bulbs of Krause, in BMS patients.

There are important limitations to consider when interpreting findings from this study. Antibody immunoreactivity might not recapitulate the protein expression pattern because of cross-reactivity with other epitopes. Future studies should compare results with antibodies against other protein epitopes, analyze RNA expression, and test functional expression of TRP channels in oral tissues. A second limitation is that tissue donors for tongue biopsies were primarily female. Future studies should analyze sex differences in TRP channel expression.

In summary, we describe the immunohistochemical localization of TRP channels throughout healthy human oral epithelium. Oral TRP channels are poised to take part in a myriad of functions including epithelial integrity, epithelial development, response to injury, thermoception, nociception, and flavor construction. Future studies are needed to parse out roles for TRP channels in oral pathologies.

## Synthesis

Reviewing Editor: Anna Menini, SISSA (International School of Advances Studies) Decisions are customarily a result of the Reviewing Editor and the peer reviewers coming together and discussing their recommendations until a consensus is reached. When revisions are invited, a fact-based synthesis statement explaining their decision and outlining what is needed to prepare a revision will be listed below. The following reviewer(s) agreed to reveal their identity: Sue Kinnamon.

Both Reviewers agree that little is known about the expression patterns of TRP channels in human tissues and that this study could add important information. However, both Reviewers are very concerned about the specificity of some antibodies that should be validated. When blocking peptides are not available, authors should for example report the test of antibodies in heterologous expression systems. Another major concern is the absence of quantification that should be added by performing image analysis. Please, include appropriate revisions to the manuscript to address the specific concerns of each Reviewer listed below:

Reviewer #1

This manuscript describes the location of transient receptor potential channel members in the human oral cavity. These channels play important roles in mechanosensation, thermosensation and chemosensation in mammals and thus a catalog of their distribution would be an important contribution to understanding their specific functions, particularly in humans where little is known. Biopsies of tongue and palate epithelia were obtained from consenting adult humans and their distribution was assessed with immunocytochemistry and confocal imaging. The results suggest that TrpA1, TrpV1, TrpV3, TrpV4, and TrpM8 are broadly expressed in human tongue and palate and the expression pattern correlates with expression in rodent tissues. Yet concerns remain as discussed below.

Major:

1. P. 7 and Fig. 1. It is always difficult validating specificity of antibodies in human tissue, but the authors were able to use antigen-block to validate expression of TrpA1 and TrpV3. It is surprising that antigens were not available for TrpV4, TrpM8, TrpV1 and TrpV4. Validation of these antibodies would significantly strengthen their paper, especially for channels that are broadly expressed in the epithelium.

2. P. 20, Fig. 2. Why no expression shown in tongue mucosa?

3. P. 24, Fig. 4C. It is not clear from the figure shown that epithelial cells near the fungiform taste buds express TrpA1-- the expression pattern appears more like nerve fibers, even though III tubulin did not appear to label. An expanded view of the taste bud would be helpful to clarify the source of this label.

4. Fig. 4-1. The co-expression of CD45 and TrpA1 is difficult to resolve due to the low resolution of the figure. A higher magnification of B and D would be more convincing. One cannot see that the yellow and blue are in the same cells.

5. P. 25, Fig. 5. Please make the purple arrows a lighter color-- they are very difficult to see on the black background. In Fig. 5B, please show an expanded view of the taste bud. It is very difficult to resolve the staining in the low mag view. For example, it is difficult to tell if the TrpM8 staining is in taste cells or nerve fibers near the purple arrow.

6. P. 27, Fig. 6B. Need an expanded view of the taste bud. It is very difficult to see the TrpV1 label inside the taste bud. Whether TrpV1 fibers enter fungiform papillae is an important question in the field since it would imply possible trigeminal modulation of taste function.

7. P. 29, Fig. 7. The summary slide is very nice, except more detail needs to be given. In the top row, what is the color significance of the dark pink and peach? These need to be included in the legend next to the figure. Also, in the same row, it is impossible to tell the difference between the NFH+ and - Neurons. Black and purple are too close to distinguish easily. Also, in the 3rd row, there are dark red as well as pink fibers-- significance? Finally, in the last row, what do the X’s represent? Again, the colors of the TrpA1, CD45+ cell and the TrpA1+ cells are hard to distinguish.

Minor:

p. 3, line 10. Receptor should be inserted after sensory

p. 5, first line of Tissue collection. Table 1 includes tongue back but sentence only refers to tongue front. Where was the epithelium taken from the back of the tongue?

p.8, 2nd paragraph, line 7. The sentence beginning “Within fungiform papillae” does not make sense.

Reviewer #2

In this study the authors investigated the expression pattern of sensory TRP channels in the human oral mucosa. The results are potentially interesting, but I belief that several aspects need correction or clarification.

In the Introduction:

1.- I strongly recommend updating the literature on the properties of the TRP channels given in the first paragraph of the introduction. I have nothing against referring to original studies, but there are certainly much newer reviews that can help the reader find a much more complete and recent information about the aspects touched by the authors.

In the Results:

2.- The first paragraph states that the specificity of antibodies was tested, but no quantitative results are given. In addition, one could wonder whether the specificity tested in human foreskin is valid for the oral mucosa.

3.- The results are presented in a qualitative way. For a study that fully focusses in anatomical localization, rigorous image analyses providing scores of colocalization and statistics should be performed to back up the conclusions. Virtually all statements give rather vague information and are not justified. For instance, just to mention a few:

- “In filiform papilla, some intraepithelial nerve fibers also extend into TRPV4+ lamina (Figure 3C).” What does “some” mean?

- “TRPA1 antibodies showed robust immunolocalization in the hard palate and tongue, with the highest density in lamina propria cells (Figure 4).”

What does “robust immunolocalization” and “highest density” mean in terms of numbers?

- “Co-staining with an antibody against CD45, a marker for immune cells, revealed that many TRPA1+ lamina propria and epithelial cells were CD45+ (Figure 4-1).”

What does “many” mean?

It is strongly recommended that all result statements are backed up with actual quantification and statistics.

In the Discussion:

4.- It is stated that “TRP channels are widely expressed in the oral cavity and subserve a variety of functions including taste transduction, somatosensation and stress responses.” This is true for taste transduction in mice, but can this general statement be extended to humans? It is recommended that careful distinction is made along the text when comparing to data obtained in mice.
