# Supplementary figures and images for: Localization of TRP Channels in Healthy Oral Mucosa from Human Donors
Source: eNeuro. 2022 Dec 21;9(6):ENEURO.0328-21.2022. doi: 10.1523/ENEURO.0328-21.2022 (PMC9797210; doi:10.1523/ENEURO.0328-21.2022)

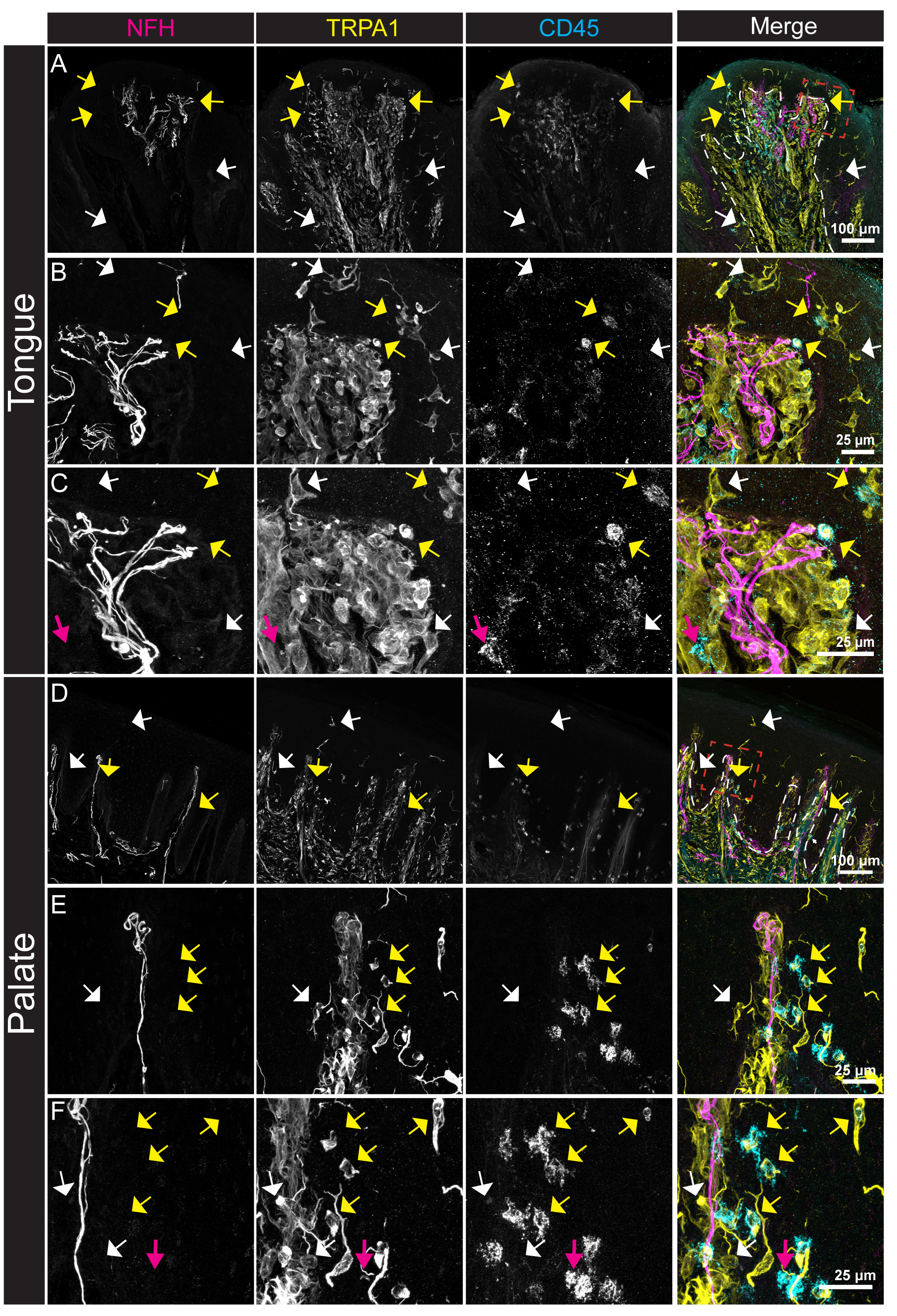

Supplement: Extended Data Figure 4-1 — TRPA1 immunoreactivity co-expresses with CD45 in tongue and hard palate rugae. Left column, NFH antibody (myelinated neurons). Second column, Anti-TRPA1 antibody. Third column, Anti-CD45 antibody (immune cells). Right column, Merge with TRP immunoreactivity in yellow, CD45 immunoreactivity in cyan, NFH immunoreactivity in magenta. Dashed line indicates epithelial-lamina propria border. A, TRPA1 was broadly expressed throughout lamina propria cells and some cells in epithelial layer of tongue. Co-expression of TRPA1 and CD45 was identified in some epithelial cells (yellow arrows). TRPA1+ cells that did not co-express CD45 (white arrows) were also found. Red box shows region in B. B, A higher magnification view of A. C, An expanded view of B. Magenta arrow denotes CD45+ cell that does not overlap with TRPA1 immunoreactivity. D, TRPA1 shows a similar pattern of expression in the hard palate as in tongue. Both cells that co-expressed CD45 (yellow arrows) and those that did not (white arrows) were identified. E, Higher magnification view of D. F, Expanded view of E. Magenta arrow denotes CD45+ cell that does not overlap with TRPA1 immunoreactivity. Download Figure 4-1, TIF file. [file enu-eN-NWR-0328-21-s02.tif]

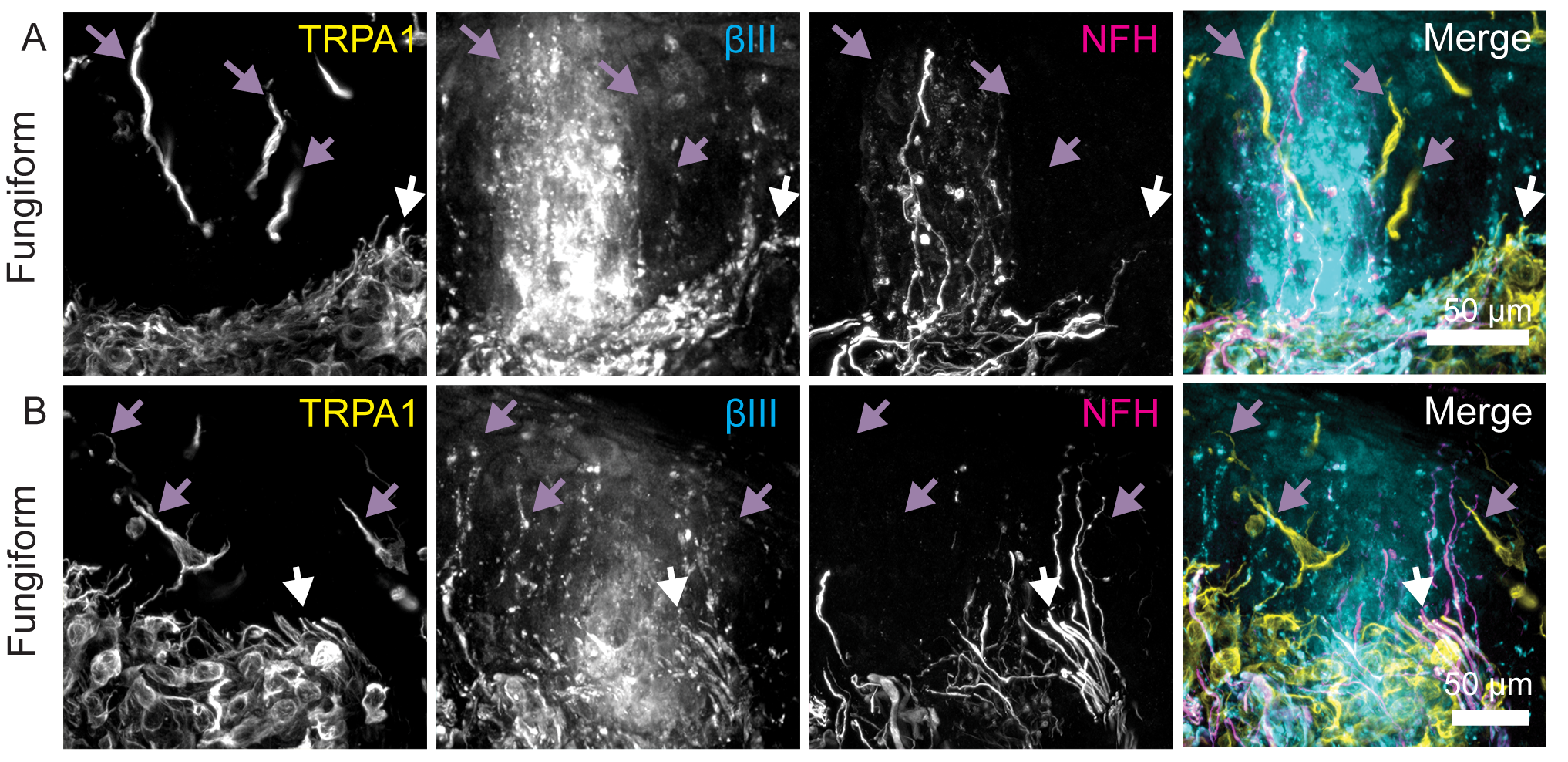

Supplement: Extended Data Figure 4-2 — TRPA1 immunoreactivity does not colocalize with neuronal fibers in the taste bud. Expanded views of taste buds in Figure 4 are shown. Left column, Anti-TRPA1 antibody. Second column, βIII tubulin antibody (all neurons). Third column, Neurofilament Heavy antibody (myelinated neurons). Right column, Merge with TRP immunoreactivity in yellow, βIII immunoreactivity in cyan, NFH immunoreactivity in magenta. A, TRPA1 immunoreactivity was identified in large-diameter fibrous processes in the taste bud (purple arrows). This expression did not colocalize with βIII-tubulin or NFH, suggesting that it is non-neuronal. Adjacent to the taste bud, a small fiber was identified with co-expression of βIII-tubulin (white arrow), suggesting it is neuronal. B. A second taste bud was assessed. Similarly, large processes that were TRPA1 immunoreactive did not co-express βIII-tubulin or NFH (purple arrows); however, small processes were identified near the basement membrane with colocalization of βIII-tubulin and TRPA1 immunoreactivity (white arrow). Download Figure 4-2, TIF file. [file enu-eN-NWR-0328-21-s03.tif]
